# The Enigma of Large-Sized, Multiple Tuberous Xanthomas: A Report of a Rare Case

**DOI:** 10.7759/cureus.97117

**Published:** 2025-11-17

**Authors:** Karthik D., Pawan Kumar, Ramesh L. J.

**Affiliations:** 1 Sports Medicine and Arthroplasty, Manipal Hospital, Bengaluru, IND; 2 Orthopedics, Dr. Chandramma Dayanda Sagar Institute of Medical Education and Research, Bengaluru, IND

**Keywords:** cardiovascular effects, familial hypercholesterolemia, foam cells, tuberous xanthomas, xanthomas

## Abstract

This case report presents a rare case of multiple xanthomas in a 20-year-old man, highlighting the clinical implications and underlying health concerns associated with such lesions. The patient exhibited multiple large tuberous xanthomas that developed over several years and were associated with familial hypercholesterolemia and valvular heart disease. A comprehensive diagnostic workup revealed significantly elevated cholesterol levels, echocardiographic findings consistent with valvular dysfunction, and characteristic xanthomatous lesions on imaging and histopathological examination. The patient underwent a multifaceted treatment approach, including dietary modifications, pharmacotherapy, and surgical excision of the lesions, to alleviate discomfort and improve quality of life. The findings underscore the necessity of heightened awareness among healthcare providers regarding the potential systemic implications of xanthomas, advocating for early diagnosis and intervention to mitigate associated cardiovascular risks. The limitations of this study include a lack of genetic testing, which could further elucidate the pathophysiology of the condition. This report emphasizes the importance of a holistic treatment strategy that combines surgical and medical management to address both the physical and psychological burdens of this condition while preventing recurrence.

## Introduction

Multiple xanthomas are uncommon dermatological manifestations that often serve as cutaneous markers of underlying systemic disease [1]. The presence of widespread xanthomatous lesions in a single patient warrants careful evaluation, as they may signify significant metabolic or cardiovascular pathology [2,3]. This case report highlights an unusual presentation of disseminated xanthomas, which not only adds to the spectrum of clinical variability but also underscores the importance of maintaining a high index of suspicion among healthcare providers [4]. Awareness of such presentations is crucial for timely diagnosis, appropriate systemic evaluation, and institution of targeted management.

## Case presentation

Patient presentation: unveiling the clinical picture

**Figure 1:**
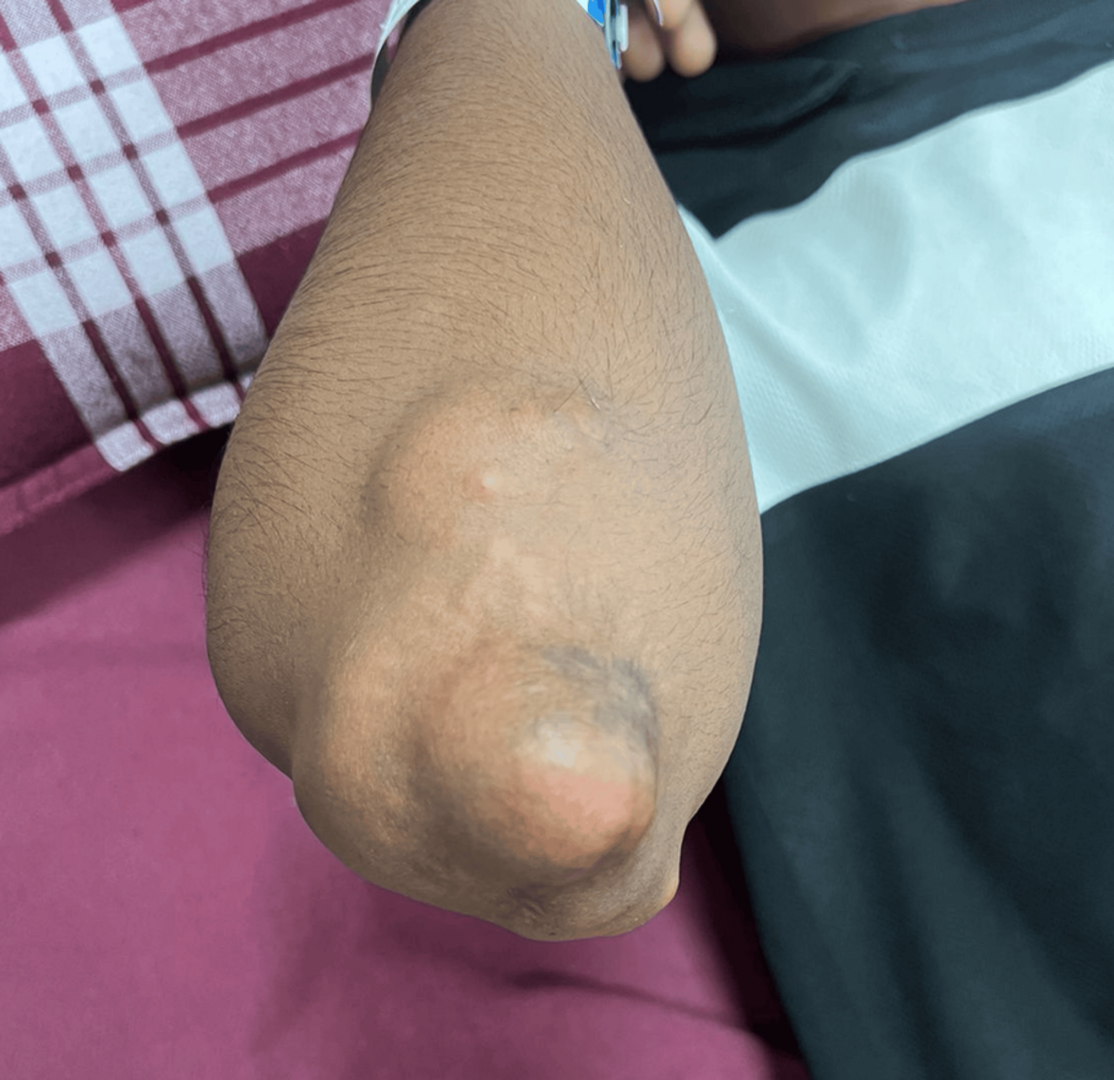
Xanthomatous lesions around the elbow.

**Figure 2:**
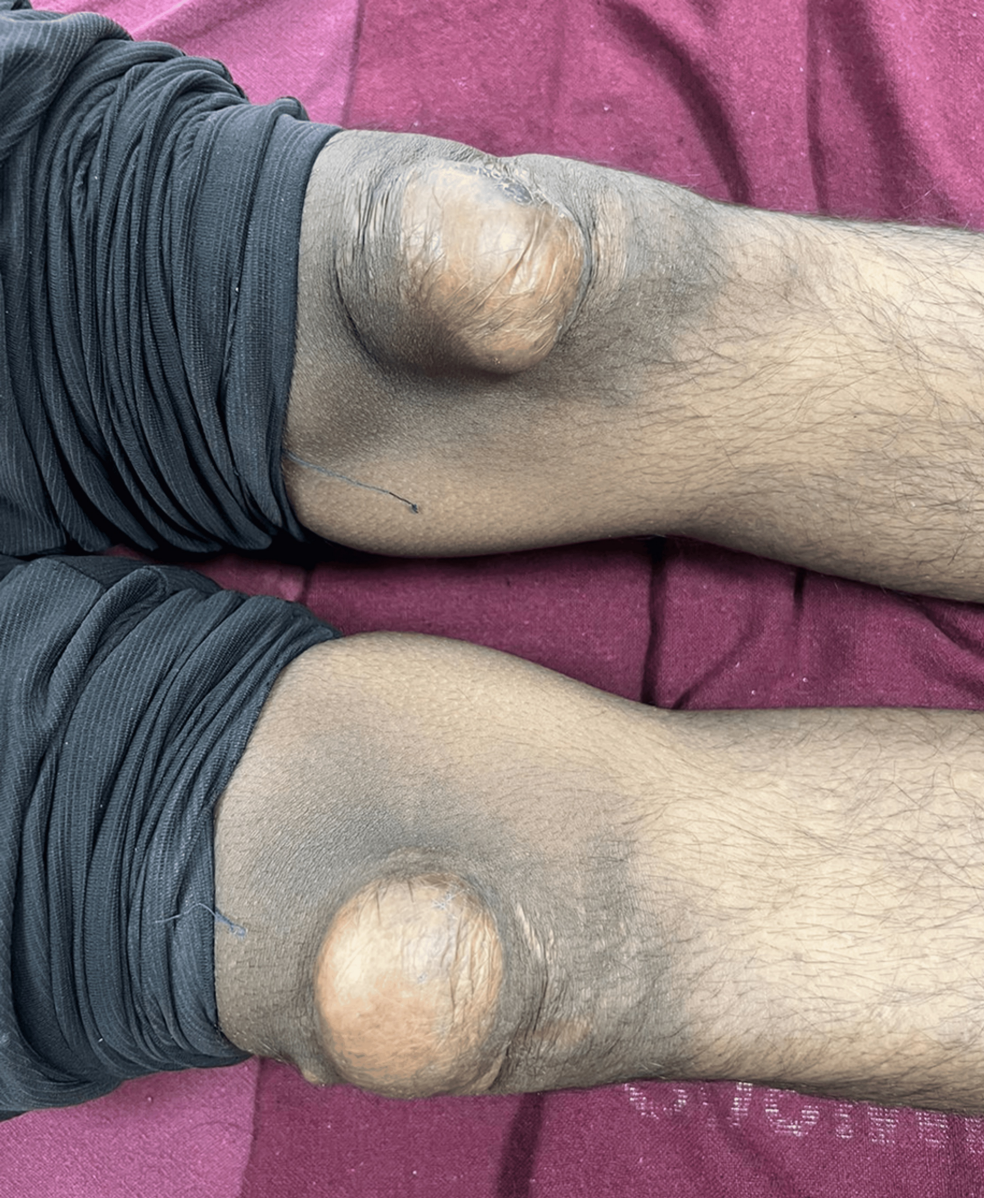
Prominent xanthomas on the anterior aspects of both knees.

A 20-year-old male patient presented to our hospital in September 2024 with multiple masses over the dorsum of the elbows, knees, and buttocks. On clinical examination, the size of the masses varied between 6 × 4 × 4 cm (over the dorsum of the elbows bilaterally), 7 × 3 × 4 cm (over the dorsal aspect of the bilateral knee), and 9 × 4 × 3 cm (over the buttock). The lesions were originally asymptomatic; they appeared at 12 years of age and progressively increased in size and extent. The patient had symptoms of discomfort and pain in the elbows and buttocks (as shown in Figures 1, 2), which were due to the large size of the masses, and had difficulty sitting due to their presence in the buttocks. His hygiene was also affected, and he felt self-conscious about wearing half-sleeve shirts. The patient was diagnosed with rheumatic heart disease with valvular heart disease (mild mitral regurgitation and moderate aortic stenosis) at 16 years of age and was on treatment for the same.

Diagnostic workup: deciphering the underlying cause

**Figure 3:**
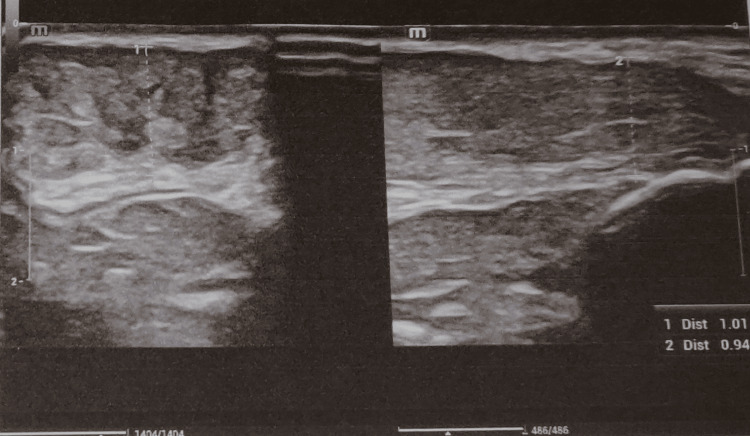
Ultrasound imaging revealed no tendon infiltration, thus differentiating it from a tendinous xanthoma.

The biochemical evaluation of the lipid profile was significantly elevated (three times the normal value). Significantly elevated low-density lipoprotein (LDL) (two times the normal value). The values of high-density lipoprotein (HDL) and triglycerides were normal. An echocardiogram revealed mild mitral regurgitation and moderate aortic stenosis. Ultrasonography of the swellings revealed xanthomas with no adjacent tendon infiltrations (Figure 3).

**Figure 4:**
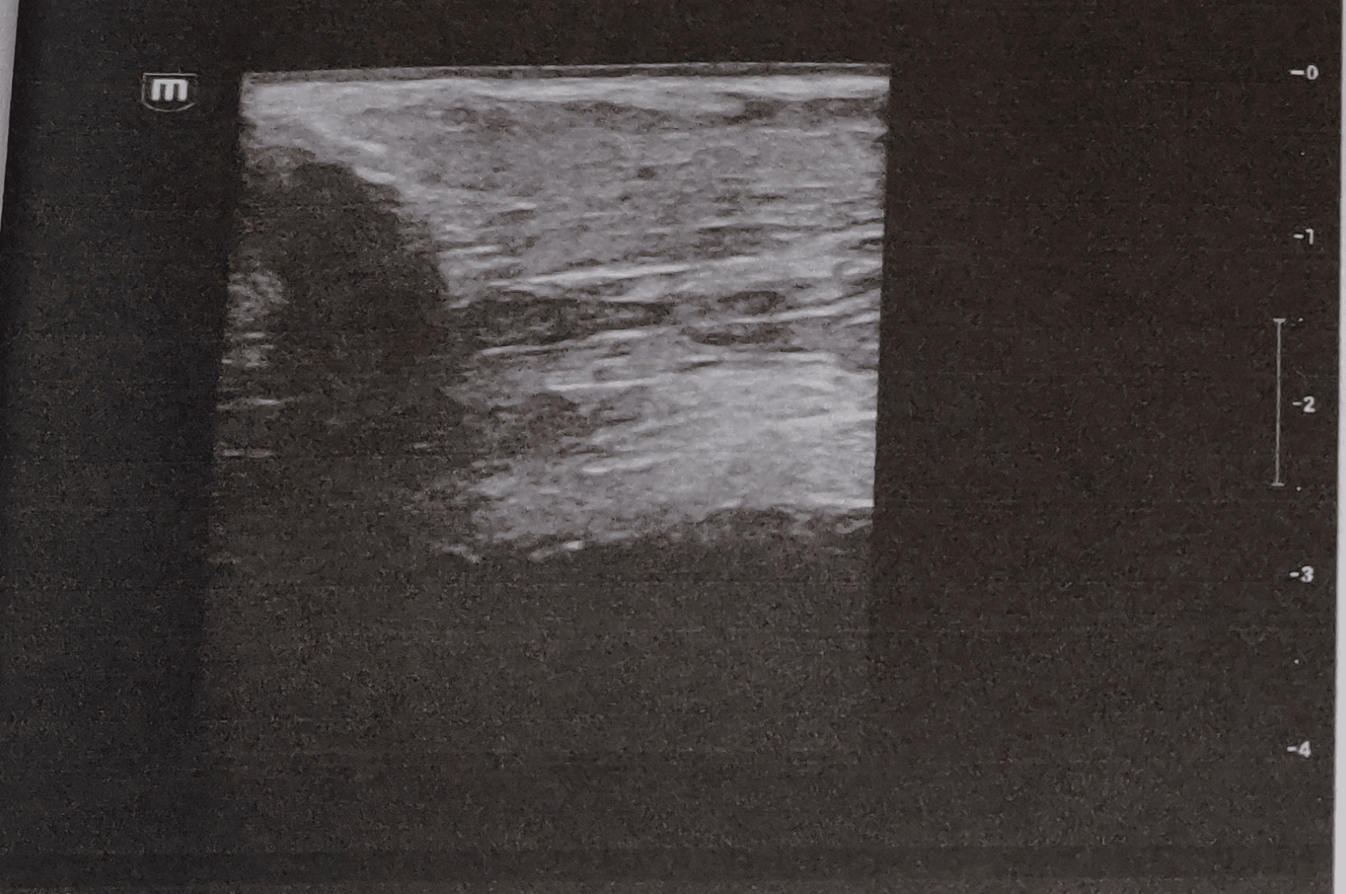
Intimal thickening on carotid Doppler ultrasound.

Carotid artery Doppler imaging revealed atheromatous plaque formation and intima-media thickening (Figure 4).

**Figure 5:**
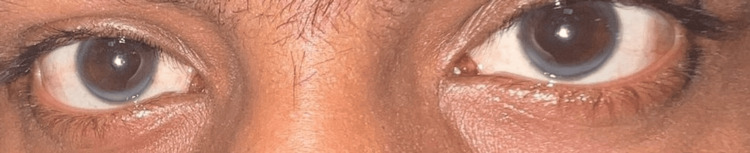
Corneal arcus in a third-decade individual should prompt evaluation for hyperlipidemia.

Ocular examination showed arcus senilis with no significant findings on fundoscopy (as shown in Figure 5). Liver and renal function tests, coagulation profile, thyroid function test, rheumatoid factor, and anti-cyclic citrullinated peptide (anti-CCP) were all found to be within lab limits (Table 1). Ultrasound of the abdomen and pelvis revealed no significant abnormality.

**Table 1:** Laboratory values showing markedly elevated total cholesterol and LDL cholesterol. LDL: low-density lipoprotein; HDL: high-density lipoprotein; AST: aspartate transaminase; ALT: alanine transaminase; anti-CCP: anti-cyclic citrullinated peptide; INR: international normalized ratio

Parameter	Patient Value	Reference Range	Interpretation
Total Cholesterol	780 mg/dL	<200 mg/dL	↑↑ Markedly elevated
LDL Cholesterol	400 mg/dL	<130 mg/dL	↑↑ Severely elevated
HDL Cholesterol	48 mg/dL	40-60 mg/dL	Within normal limits
Triglycerides	140 mg/dL	<150 mg/dL	Normal
Liver Function Tests (AST/ALT)	28/32 U/L	<40 U/L	Normal
Renal Function (Creatinine)	0.9 mg/dL	0.6-1.3 mg/dL	Normal
Thyroid-Stimulating Hormone	2.0 µIU/mL	0.4-4.0 µIU/mL	Normal
Rheumatoid Factor	Negative	Negative	Normal
Anti-CCP Antibodies	Negative	Negative	Normal
Coagulation Profile (INR)	1	0.9-1.2	Normal

In this case, however, all the swellings were greater than 2 cms, which makes it an atypical presentation for tuberous xanthoma. The patient gave the history of his mother and maternal grandmother dying at a young age due to suspected cardiac causes. The present study reports the case of a 20-year-old male patient with multiple masses who presented with multiple large tuberous xanthomas within various dermal tissues secondary to familial hypercholesterolemia (FH) with valvular heart disease.

Treatment and management: a multifaceted approach

In terms of diet, the patient was subsequently advised to follow a strict vegetarian diet low in saturated fat and cholesterol. The patient was treated with a combined treatment regimen of atorvastatin (20 mg/day) and Ecosprin (75 mg/day) to correct the hyperlipidemia and reduce the risk of cardiovascular events.

**Figure 6:**
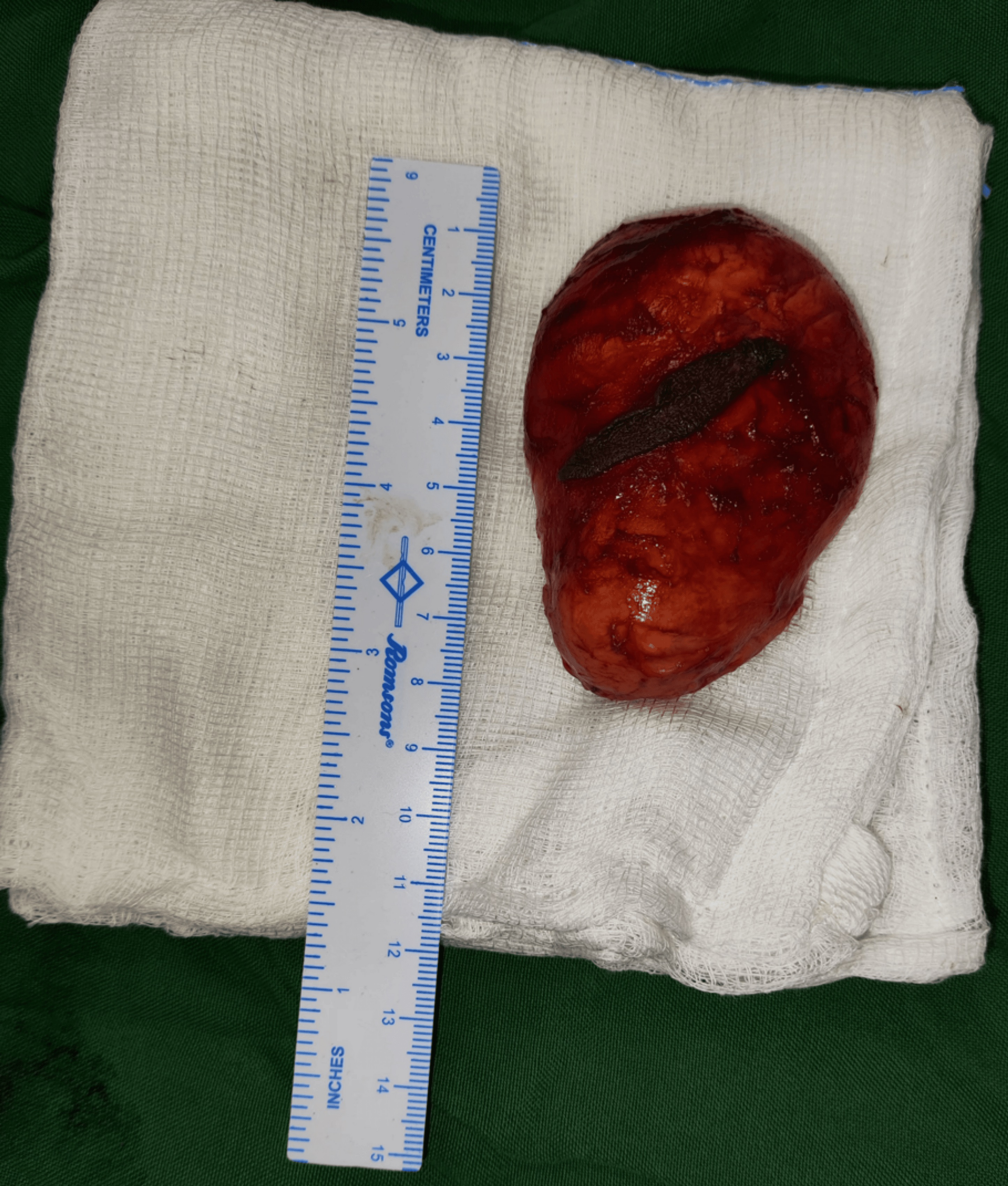
Resected specimens were as large as 7 × 6 cm.

The lesions caused significant discomfort to the patient in his activities of daily living, and he was significantly concerned about the cosmetic discomfort caused by the lesions. Surgical excision of the masses over the elbows, knees, and buttocks was performed. Resected tissues were sent for histopathological examinations (as shown in Figure 6).

**Figure 7:**
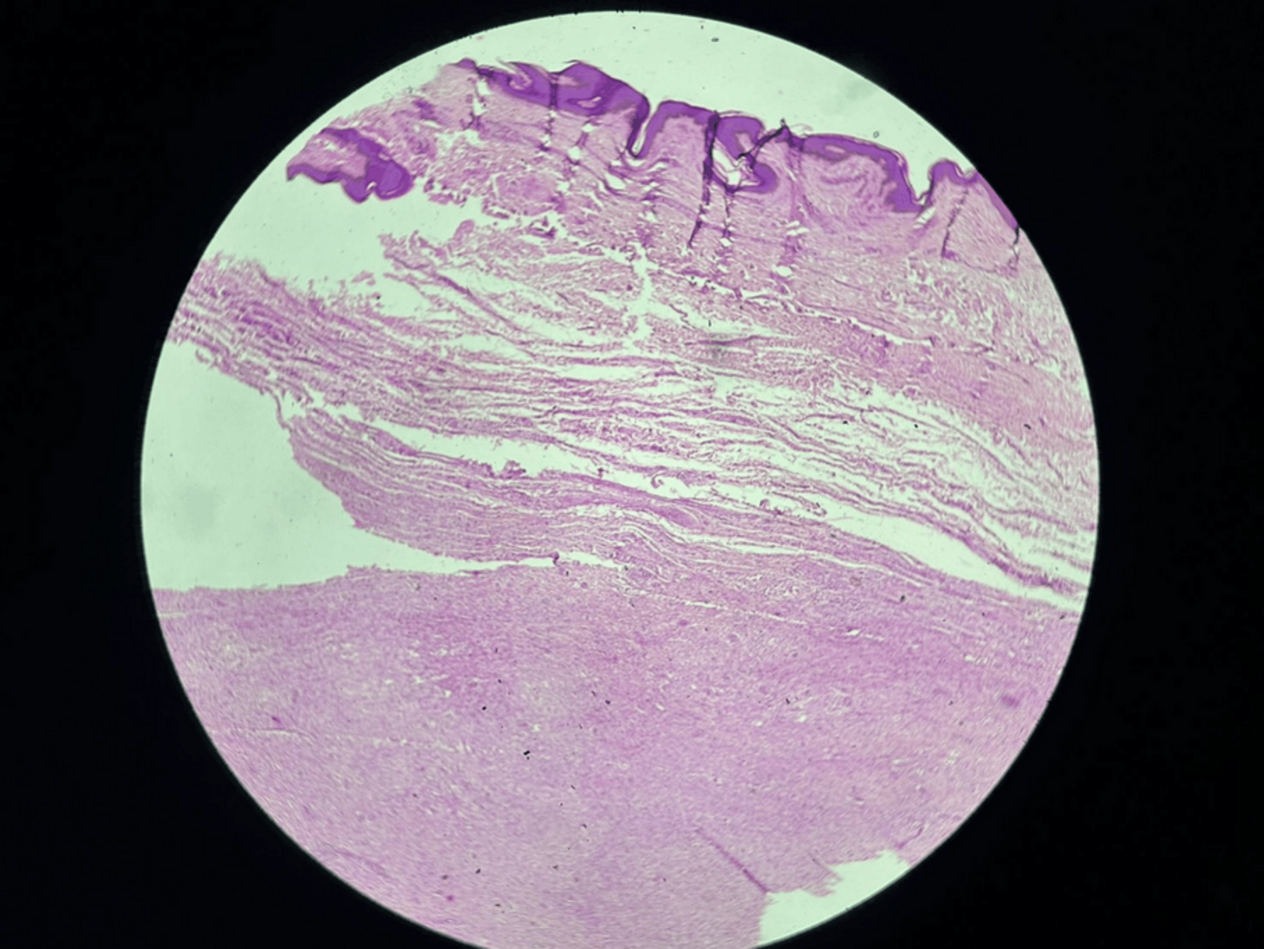
Microscopy image at 10× showing the dermis.

**Figure 8:**
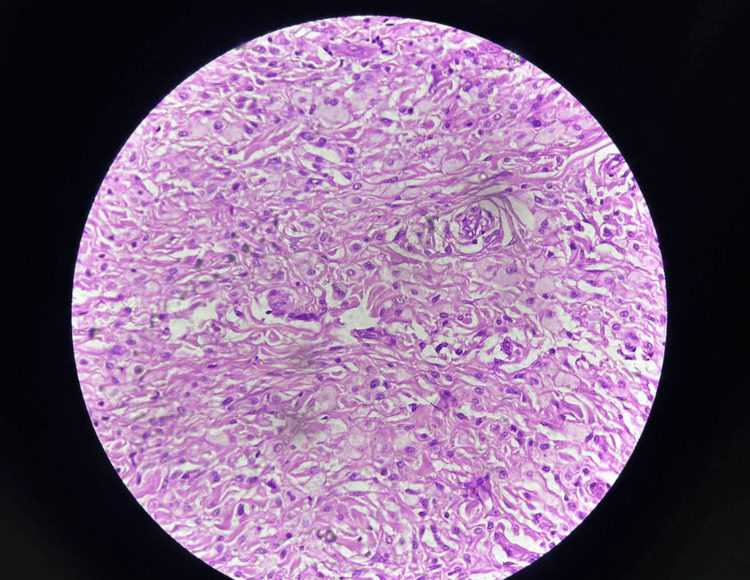
Microscopy image at 40x.

Microscopic examination of the surgical specimens revealed sheets of xanthomatous cells with abundant foamy cytoplasm against a background of degenerated collagenous stroma (as shown in Figures 7, 8). These findings further confirmed the diagnosis of FH with multiple xanthomas.

At one-year follow-up, we noticed no recurrence of any lesions and a well-controlled lipid profile (follow-up total cholesterol: 156 mg/dL and LDL cholesterol: 96 mg/dL).

## Discussion

Tuberous xanthomas are infrequent dermatological manifestations that typically signify an underlying metabolic disorder, most notably FH. These lesions are generally small, measuring less than 2 cm, and are found on extensor surfaces subjected to repetitive trauma, such as the elbows, knees, and buttocks [5,6]. The present case is notable for the unusually large size and multiplicity of lesions, which are rarely documented in the literature and highlight the aggressive phenotype of lipid metabolism disorders in young patients [7].

The pathogenesis of tuberous xanthomas is associated with the deposition of LDL cholesterol in macrophages within the dermis and subcutaneous tissue, leading to the accumulation of foam cells [5]. In patients with homozygous or severe heterozygous FH, persistently elevated LDL-C levels accelerate this process, resulting in cutaneous manifestations and systemic vascular complications, including premature coronary artery disease and valvular dysfunction [6,7]. The co-occurrence of valvular heart disease in this patient underscores the systemic implications of untreated hypercholesterolemia in patients with FH. While dietary modification and statin therapy remain the cornerstones of medical management, surgical excision is often pursued for symptomatic or cosmetically disfiguring lesions. However, recurrence following excision is well documented, particularly in patients with poorly controlled lipid levels [8,9]. Thus, the combination of local surgical management and long-term lipid-lowering pharmacotherapy is considered optimal [6]. In resource-limited settings, the lack of access to advanced therapies, such as PCSK9 inhibitors, poses a challenge in adequately reducing cardiovascular risk. Another limitation in the management of this case was the absence of genetic testing, which has become increasingly important for confirming FH, guiding family screening, and tailoring therapy. Recent advances in next-generation sequencing have facilitated the early detection of pathogenic mutations, allowing for preemptive risk stratification of at-risk relatives. In the present case, the family history of premature cardiac death strongly suggested inherited dyslipidemia, and genetic evaluation could have provided valuable prognostic and preventive insights.

This case highlights several important clinical insights. First, xanthomas should not be dismissed as mere cosmetic lesions because they serve as visible markers of severe systemic disease. Second, multidisciplinary care involving dermatologists, cardiologists, and geneticists is essential for ensuring optimal outcomes. Finally, there is a need for greater awareness of FH in young individuals presenting with xanthomas, as early diagnosis and intervention can significantly reduce the morbidity and mortality associated with premature cardiovascular events [5,6,9].

## Conclusions

In cases of large and painful xanthomas that impede mobility, surgical intervention may be necessary. However, studies have noted a high rate of recurrence after surgery. To lower the chances of recurrence, for extensive tendinous and tuberous xanthomas, a combination of local surgical excision and subsequent cholesterol-lowering therapy is considered the most effective approach. The present case sheds light on the importance of prompt cytopathological diagnosis of xanthomatous lesions, as it can help prevent morbidity and mortality due to associated premature adverse cardiovascular and cerebrovascular events if left undiagnosed. This case also highlights how the successful removal of the lesions and aggressive medical management of the lipid profile led to improving the overall quality of life and successfully reducing the risk of cardiac complications.
